# 2-[2-(4-Meth­oxy­phen­yl)-2-oxoeth­yl]malononitrile

**DOI:** 10.1107/S1600536811026961

**Published:** 2011-07-13

**Authors:** Mi Lian, Tianli Chen, Yanping Zhu

**Affiliations:** a Key Laboratory of Pesticide and Chemical Biology of the Ministry of Education, College of Chemistry, Central China Normal University, Wuhan 430079, People’s Republic of China; b The 61081 group of PLA, Beijing 100094, People’s Republic of China

## Abstract

The title compound, C_12_H_10_N_2_O_2_, was obtained unintentionally during the synthesis of 2-amino-5-(4-meth­oxy­phen­yl)furan-3-carbonitrile. In the crystal, weak inter­mol­ecular C—H⋯N and C—H⋯π inter­actions link the mol­ecules into columns propagating in [010].

## Related literature

For the crystal structures of related compounds with a malononitrile fragment, see: Luo & Zhou (2006 ▶); Ohashi *et al.* (2008 ▶); Oliva *et al.* (2010 ▶).
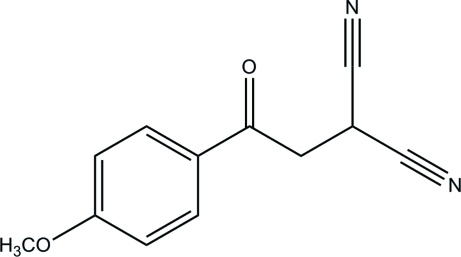


## Experimental

### 

#### Crystal data

C_12_H_10_N_2_O_2_
*M* *_r_* = 214.22
Monoclinic, 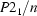

*a* = 11.9010 (13) Å
*b* = 6.4898 (7) Å
*c* = 14.4248 (16) Å
β = 100.141 (2)°
*V* = 1096.7 (2) Å^3^
*Z* = 4
Mo *K*α radiation
μ = 0.09 mm^−1^
*T* = 298 K
0.16 × 0.12 × 0.10 mm

#### Data collection

Bruker SMART CCD area-detector diffractometer
11044 measured reflections
2148 independent reflections
1693 reflections with *I* > 2σ(*I*)
*R*_int_ = 0.123

#### Refinement

*R*[*F* ^2^ > 2σ(*F* ^2^)] = 0.052
*wR*(*F* ^2^) = 0.137
*S* = 1.07
2148 reflections
146 parameters
H-atom parameters constrained
Δρ_max_ = 0.18 e Å^−3^
Δρ_min_ = −0.21 e Å^−3^

### 

Data collection: *SMART* (Bruker, 1997 ▶); cell refinement: *SAINT* (Bruker, 1997 ▶); data reduction: *SAINT*; program(s) used to solve structure: *SHELXS97* (Sheldrick, 2008 ▶); program(s) used to refine structure: *SHELXL97* (Sheldrick, 2008 ▶); molecular graphics: *SHELXTL* (Sheldrick, 2008 ▶); software used to prepare material for publication: *SHELXTL*.

## Supplementary Material

Crystal structure: contains datablock(s) I, global. DOI: 10.1107/S1600536811026961/cv5094sup1.cif

Structure factors: contains datablock(s) I. DOI: 10.1107/S1600536811026961/cv5094Isup2.hkl

Supplementary material file. DOI: 10.1107/S1600536811026961/cv5094Isup3.cml

Additional supplementary materials:  crystallographic information; 3D view; checkCIF report

## Figures and Tables

**Table 1:** Hydrogen-bond geometry (Å, °) *Cg* is the centroid of the C1–C6 ring.

*D*—H⋯*A*	*D*—H	H⋯*A*	*D*⋯*A*	*D*—H⋯*A*
C9—H9*B*⋯N1^i^	0.97	2.55	3.380 (2)	143
C10—H10⋯*Cg*^ii^	0.98	2.56	3.411 (1)	145

Symmetry codes: (i) 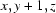; (ii) 
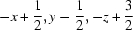
.

**Fig. 1.**
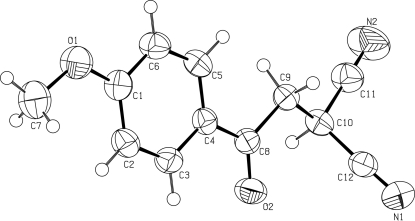
A view of (I), showing the atom-labelling scheme, with displacement ellipsoids drawn at the 50% probability level.

## supplementary crystallographic information

### Comment

The title compound (I) has been unintentionally obtained in the process of synthesis of 2-amino-5-(4-methoxyphenyl)furan-3-carbonitrile.

In (I) (Fig. 1), all bond lengths and angles are normal and comparable with those observed in related compounds (Luo & Zhou, 2006; Ohashi *et al.*, 2008; Oliva *et al.*, 2010). In the crystal structure, weak intermolecular C—H···N and C—H···π interactions (Table 1) link the molecules into columns propagated in [010].

### Experimental

To a solution of K_2_CO_3_ (2.0 equiv) in MeOH,3-iodo-1-(4-methoxyphenyl)propan-1-one (1.0 equiv) and malononitrile (2.0 equiv) were separately added. The resulting mixture was then heated at reflux for several hours (TLC monitoring). After that, the solvent was removed under reduce pressure, and added 50 mL water to the residue, then extracted with EtOAc 3 times. The organic phase was washed with saturated saline solution. Then the organic phase was dried by anhydrous Na2SO4, and removed the EtOAc under reduce pressure. The final residue was purified by column chromatography on silica gel to afford the expected target compound as a white solid.

### Refinement

All H atoms were positioned in geometrically idealized positions and constrained to ride on their parent atoms, with C—H distances in the range 0.93–0.97 Å and *U*_iso_(H) = 1.2-1.5*U*_eq_(C).

### Crystal data

**Table:**

C_12_H_10_N_2_O_2_	*F*(000) = 448
*M**_r_* = 214.22	*D*_x_ = 1.297 Mg m^−^^3^
Monoclinic, *P*2_1_/*n*	Mo *K*α radiation, λ = 0.71073 Å
*a* = 11.9010 (13) Å	Cell parameters from 2694 reflections
*b* = 6.4898 (7) Å	θ = 2.9–26.8°
*c* = 14.4248 (16) Å	µ = 0.09 mm^−^^1^
β = 100.141 (2)°	*T* = 298 K
*V* = 1096.7 (2) Å^3^	Block, colourless
*Z* = 4	0.16 × 0.12 × 0.10 mm

### Data collection

**Table:**

Bruker SMART CCD area-detector diffractometer	1693 reflections with *I* > 2σ(*I*)
Radiation source: fine-focus sealed tube	*R*_int_ = 0.123
graphite	θ_max_ = 26.0°, θ_min_ = 2.4°
phi and ω scans	*h* = −14→14
11044 measured reflections	*k* = −7→7
2148 independent reflections	*l* = −17→17

### Refinement

**Table:**

Refinement on *F*^2^	Primary atom site location: structure-invariant direct methods
Least-squares matrix: full	Secondary atom site location: difference Fourier map
*R*[*F*^2^ > 2σ(*F*^2^)] = 0.052	Hydrogen site location: inferred from neighbouring sites
*wR*(*F*^2^) = 0.137	H-atom parameters constrained
*S* = 1.07	*w* = 1/[σ^2^(*F*_o_^2^) + (0.0648*P*)^2^ + 0.034*P*] where *P* = (*F*_o_^2^ + 2*F*_c_^2^)/3
2148 reflections	(Δ/σ)_max_ < 0.001
146 parameters	Δρ_max_ = 0.18 e Å^−^^3^
0 restraints	Δρ_min_ = −0.21 e Å^−^^3^

### Special details

**Table:**

Geometry. All e.s.d.'s (except the e.s.d. in the dihedral angle between two l.s. planes) are estimated using the full covariance matrix. The cell e.s.d.'s are taken into account individually in the estimation of e.s.d.'s in distances, angles and torsion angles; correlations between e.s.d.'s in cell parameters are only used when they are defined by crystal symmetry. An approximate (isotropic) treatment of cell e.s.d.'s is used for estimating e.s.d.'s involving l.s. planes.
Refinement. Refinement of *F*^2^ against ALL reflections. The weighted *R*-factor *wR* and goodness of fit *S* are based on *F*^2^, conventional *R*-factors *R* are based on *F*, with *F* set to zero for negative *F*^2^. The threshold expression of *F*^2^ > σ(*F*^2^) is used only for calculating *R*-factors(gt) *etc*. and is not relevant to the choice of reflections for refinement. *R*-factors based on *F*^2^ are statistically about twice as large as those based on *F*, and *R*- factors based on ALL data will be even larger.

### Fractional atomic coordinates and isotropic or equivalent isotropic displacement parameters (Å^2^)

**Table:**

	*x*	*y*	*z*	*U*_iso_*/*U*_eq_	
C1	−0.03121 (14)	0.6967 (3)	0.89840 (11)	0.0469 (4)	
C2	−0.05989 (14)	0.5030 (3)	0.86164 (11)	0.0480 (4)	
H2	−0.1361	0.4645	0.8453	0.058*	
C3	0.02602 (14)	0.3670 (2)	0.84951 (11)	0.0448 (4)	
H3	0.0068	0.2371	0.8245	0.054*	
C4	0.14054 (13)	0.4210 (2)	0.87402 (10)	0.0396 (4)	
C5	0.16687 (14)	0.6144 (2)	0.91392 (11)	0.0462 (4)	
H5	0.2429	0.6514	0.9330	0.055*	
C6	0.08248 (15)	0.7514 (3)	0.92565 (12)	0.0509 (5)	
H6	0.1015	0.8805	0.9518	0.061*	
C7	−0.22564 (17)	0.8083 (3)	0.87859 (16)	0.0742 (6)	
H7A	−0.2385	0.7757	0.8126	0.111*	
H7B	−0.2686	0.9290	0.8887	0.111*	
H7C	−0.2497	0.6947	0.9130	0.111*	
C8	0.22937 (13)	0.2781 (2)	0.85246 (11)	0.0406 (4)	
C9	0.35302 (13)	0.3417 (2)	0.88084 (11)	0.0427 (4)	
H9A	0.3737	0.3396	0.9490	0.051*	
H9B	0.3625	0.4815	0.8597	0.051*	
C10	0.43241 (13)	0.1983 (2)	0.83864 (11)	0.0434 (4)	
H10	0.4024	0.1853	0.7711	0.052*	
C11	0.54808 (15)	0.2847 (3)	0.84914 (13)	0.0552 (5)	
C12	0.44048 (14)	−0.0098 (3)	0.87938 (12)	0.0489 (4)	
N1	0.45034 (15)	−0.1695 (2)	0.91202 (13)	0.0714 (5)	
N2	0.63714 (16)	0.3512 (3)	0.85611 (16)	0.0868 (6)	
O1	−0.10731 (11)	0.84605 (18)	0.91043 (10)	0.0646 (4)	
O2	0.20628 (10)	0.11578 (17)	0.81148 (9)	0.0555 (4)	

### Atomic displacement parameters (Å^2^)

**Table:**

	*U*^11^	*U*^22^	*U*^33^	*U*^12^	*U*^13^	*U*^23^
C1	0.0446 (10)	0.0516 (10)	0.0465 (9)	0.0042 (8)	0.0132 (7)	0.0010 (8)
C2	0.0366 (9)	0.0558 (10)	0.0517 (10)	−0.0036 (7)	0.0078 (7)	0.0008 (8)
C3	0.0408 (9)	0.0429 (9)	0.0509 (10)	−0.0045 (7)	0.0085 (7)	0.0012 (7)
C4	0.0362 (9)	0.0451 (9)	0.0375 (8)	−0.0014 (7)	0.0062 (6)	0.0034 (7)
C5	0.0373 (9)	0.0540 (10)	0.0466 (9)	−0.0056 (7)	0.0052 (7)	−0.0037 (7)
C6	0.0493 (11)	0.0499 (10)	0.0545 (10)	−0.0035 (8)	0.0118 (8)	−0.0097 (8)
C7	0.0472 (12)	0.0813 (14)	0.0951 (16)	0.0128 (10)	0.0147 (11)	−0.0016 (12)
C8	0.0404 (9)	0.0410 (9)	0.0393 (8)	−0.0038 (7)	0.0046 (7)	0.0044 (7)
C9	0.0385 (9)	0.0424 (9)	0.0467 (9)	−0.0003 (7)	0.0058 (7)	0.0007 (7)
C10	0.0386 (9)	0.0489 (9)	0.0425 (9)	−0.0050 (7)	0.0067 (7)	−0.0049 (7)
C11	0.0448 (11)	0.0574 (11)	0.0650 (11)	−0.0034 (8)	0.0141 (9)	−0.0071 (9)
C12	0.0408 (10)	0.0476 (10)	0.0561 (10)	−0.0033 (7)	0.0025 (7)	−0.0117 (8)
N1	0.0717 (12)	0.0465 (9)	0.0902 (13)	−0.0040 (8)	−0.0018 (9)	−0.0014 (9)
N2	0.0493 (11)	0.0921 (14)	0.1221 (17)	−0.0202 (9)	0.0236 (10)	−0.0177 (11)
O1	0.0473 (8)	0.0633 (8)	0.0844 (9)	0.0092 (6)	0.0145 (6)	−0.0115 (7)
O2	0.0450 (7)	0.0497 (7)	0.0704 (8)	−0.0058 (5)	0.0065 (6)	−0.0134 (6)

### Geometric parameters (Å, °)

**Table:**

C1—O1	1.3585 (19)	C7—H7A	0.9600
C1—C2	1.383 (2)	C7—H7B	0.9600
C1—C6	1.387 (2)	C7—H7C	0.9600
C2—C3	1.385 (2)	C8—O2	1.2153 (18)
C2—H2	0.9300	C8—C9	1.514 (2)
C3—C4	1.391 (2)	C9—C10	1.527 (2)
C3—H3	0.9300	C9—H9A	0.9700
C4—C5	1.393 (2)	C9—H9B	0.9700
C4—C8	1.480 (2)	C10—C12	1.469 (2)
C5—C6	1.374 (2)	C10—C11	1.469 (2)
C5—H5	0.9300	C10—H10	0.9800
C6—H6	0.9300	C11—N2	1.132 (2)
C7—O1	1.423 (2)	C12—N1	1.136 (2)
			
O1—C1—C2	124.91 (15)	O1—C7—H7C	109.5
O1—C1—C6	114.83 (15)	H7A—C7—H7C	109.5
C2—C1—C6	120.26 (15)	H7B—C7—H7C	109.5
C1—C2—C3	119.30 (15)	O2—C8—C4	122.46 (14)
C1—C2—H2	120.3	O2—C8—C9	119.58 (14)
C3—C2—H2	120.3	C4—C8—C9	117.94 (13)
C2—C3—C4	121.31 (15)	C8—C9—C10	111.48 (13)
C2—C3—H3	119.3	C8—C9—H9A	109.3
C4—C3—H3	119.3	C10—C9—H9A	109.3
C3—C4—C5	118.08 (15)	C8—C9—H9B	109.3
C3—C4—C8	119.53 (14)	C10—C9—H9B	109.3
C5—C4—C8	122.30 (14)	H9A—C9—H9B	108.0
C6—C5—C4	121.19 (15)	C12—C10—C11	108.40 (14)
C6—C5—H5	119.4	C12—C10—C9	113.72 (13)
C4—C5—H5	119.4	C11—C10—C9	111.12 (13)
C5—C6—C1	119.79 (16)	C12—C10—H10	107.8
C5—C6—H6	120.1	C11—C10—H10	107.8
C1—C6—H6	120.1	C9—C10—H10	107.8
O1—C7—H7A	109.5	N2—C11—C10	179.2 (2)
O1—C7—H7B	109.5	N1—C12—C10	177.78 (18)
H7A—C7—H7B	109.5	C1—O1—C7	118.68 (14)
			
O1—C1—C2—C3	177.46 (15)	C3—C4—C8—C9	−179.59 (14)
C6—C1—C2—C3	−2.1 (2)	C5—C4—C8—C9	3.9 (2)
C1—C2—C3—C4	0.4 (2)	O2—C8—C9—C10	8.6 (2)
C2—C3—C4—C5	1.9 (2)	C4—C8—C9—C10	−169.81 (12)
C2—C3—C4—C8	−174.84 (14)	C8—C9—C10—C12	−69.54 (17)
C3—C4—C5—C6	−2.4 (2)	C8—C9—C10—C11	167.82 (13)
C8—C4—C5—C6	174.22 (14)	C12—C10—C11—N2	111 (14)
C4—C5—C6—C1	0.7 (3)	C9—C10—C11—N2	−124 (14)
O1—C1—C6—C5	−178.01 (15)	C11—C10—C12—N1	9(5)
C2—C1—C6—C5	1.6 (3)	C9—C10—C12—N1	−115 (5)
C3—C4—C8—O2	2.0 (2)	C2—C1—O1—C7	−3.7 (2)
C5—C4—C8—O2	−174.53 (15)	C6—C1—O1—C7	175.89 (16)

### Hydrogen-bond geometry (Å, °)

**Table:**

Cg is the centroid of the C1–C6 ring.

**Table:**

*D*—H···*A*	*D*—H	H···*A*	*D*···*A*	*D*—H···*A*
C9—H9B···N1^i^	0.97	2.55	3.380 (2)	143
C10—H10···Cg^ii^	0.98	2.56	3.411 (1)	145

Symmetry codes: (i) *x*, *y*+1, *z*; (ii) −*x*+1/2, *y*−1/2, −*z*+3/2.
